# A Comparative Analysis of Surgical Results: The hinotori™ Robotic System Versus the da Vinci® Surgical System in Simple Hysterectomy for Benign Uterine Diseases

**DOI:** 10.7759/cureus.78975

**Published:** 2025-02-13

**Authors:** Shinichi Togami, Furuzono Nozomi, Chikako Nagata, Mika Mizuno, Hiroaki Kobayashi

**Affiliations:** 1 Department of Obstetrics and Gynecology, Faculty of Medicine, Kagoshima University, Kagoshima, JPN

**Keywords:** benign uterine diseases, hinotori, robotic surgery, simple hysterectomy, surgical robot system

## Abstract

Introduction

Simple hysterectomy (SH) is a frequently performed surgery for treating benign uterine conditions. The da Vinci® Surgical System (dVSS; Intuitive Surgical, Inc., Sunnyvale, California, US) received FDA approval in 2005 for robot-assisted minimally invasive gynecological surgeries. In Japan, insurance coverage for dVSS applications in benign uterine conditions, such as uterine myomas, began in 2018, driving a sharp rise in robotic procedures. The hinotori™ Surgical Robot System (hinotori; Medicaroid Corporation, Kobe, Japan), developed in Japan, was introduced in 2020. This study compares the operative results of SH performed by the hinotori and the dVSS.

Methods

A retrospective analysis was conducted on 40 patients who underwent SH for benign disease at Kagoshima University Hospital between 2017 and 2024. Surgical parameters, including operation times, cockpit or console times, and complication rates, were compared across the two systems.

Results

Median operation times and cockpit or console times for the hinotori were comparable to those of the dVSS, with no statistical differences observed. However, roll-in to cockpit or console initiation was significantly longer for the hinotori (P = 0.024) while hospital stays were notably shorter. Conversion rates to laparotomy and operative complications showed no significant differences among the groups.

Conclusions

The hinotori demonstrated surgical results equivalent to the dVSS, supporting its safety and effectiveness in SH for benign uterine conditions. Further studies are recommended to confirm these results.

## Introduction

Simple hysterectomy (SH) is one of the usually performed procedures for managing benign uterine conditions, with approximately 500,000 cases conducted annually in the United States [1]. The da Vinci® Surgical System (dVSS) (Intuitive Surgical, Inc., Sunnyvale, California, US) was approved in the United States in 2005 for minimally invasive gynecological procedures such as hysterectomy [2]. Globally, over one million robotic procedures are performed annually [3]. The benefits of minimally invasive robotic techniques include shorter recovery times, reduced intraoperative blood loss, and decreased postoperative discomfort. Robotic-assisted surgeries enhance precision through three-dimensional visualization, the flexibility of multi-jointed instruments, and tremor elimination. In Japan, insurance coverage for dVSS in benign gynecological surgeries, such as uterine myoma treatment, became available in 2018, leading to its widespread adoption for these conditions.

The hinotori™ Surgical Robot System (hinotori), developed by Medicaroid Corporation (Medicaroid Corporation, Kobe, Japan), represents a significant advancement in robotic surgery. Covered by the national insurance in 2020 for urological surgeries, its applications expanded to include gynecological and gastrointestinal procedures by late 2022. Early reports have documented its use in gynecological operations, including simple hysterectomies for benign uterine diseases [4]. While the utility of dVSS in SH has been extensively evaluated and compared with laparoscopic approaches [5], evidence specific to the hinotori remains scarce. To address this gap, this study aims to compare the surgical results of SH performed using the hinotori with those achieved using the dVSS.

## Materials and methods

Study design and setting

This retrospective study, conducted at Kagoshima University Hospital, evaluated patients who underwent robot-assisted SH for benign uterine diseases between 2017 and 2024. The study adheres to the Strengthening the Reporting of Observational Studies in Epidemiology (STROBE) guidelines and follows the PICO (Population, Intervention, Comparison, Outcome) format. Ethical approval was obtained from the Kagoshima University Institutional Review Board (Approval No. 220155), and all participants provided written informed consent before enrollment.

Patient selection using the PICO framework

Population (P)

This study included a total of 40 patients who underwent robot-assisted SH for benign uterine diseases. The inclusion and exclusion criteria were as follows.

Inclusion criteria: Patients diagnosed with uterine myoma, adenomyosis, endometrial polyp, endometrial hyperplasia, or cervical intraepithelial neoplasia/dysplasia and patients undergoing robot-assisted SH at Kagoshima University Hospital were included in the study.

Exclusion criteria: Patients who underwent robot-assisted sacrocolpopexy or patients diagnosed with invasive cancer postoperatively were excluded from the study.

Intervention (I): Surgical Systems Used

Robotic Surgical Systems: Surgeries were performed using two different robotic platforms: da Vinci® Xi Surgical System and hinotori™ Surgical Robot System.

Both systems feature integrated robotic platforms with multiple robotic arms designed to enhance precision and dexterity. The key distinctions are included in Table 1.

**Table 1:** Features of the da Vinci® Xi and hinotori™ surgical robot systems

Feature	da Vinci® Xi	hinotori™ SRS
Robotic Arms	7-axis articulated arms	8-axis articulated arms
Docking Mechanism	Requires trocar docking (remote center pivot)	Free-docking mechanism (pivot point system)
Visualization	3D high-definition	3D high-definition
Control Mechanism	Finger and hand-controlled clutch	Finger and hand-controlled clutch
Instrument Compatibility	Force Bipolar, Monopolar, Vessel Sealer	Bipolar, Monopolar, Universal Grasper
Network Support	Not applicable	Medicaroid Intelligent Network System

All hinotori procedures were conducted using Medicaroid's Intelligent Network System, and surgeries were performed under Institutional Review Board approval.

Comparison (C): Surgical Procedure

Surgical indications and allocation of robotic systems: The choice of robotic system was based on surgical slot availability and timing of introduction, with no stratification based on BMI or disease type. Four gynecologic oncologists, all board-certified specialists, performed the surgeries. Two of them were certified in advanced laparoscopic surgery. Each surgeon performed 20-25 robotic SH cases annually, ensuring comparable expertise between both systems.

Standardized surgical protocol: The same protocol was followed for both robotic systems. It included general anesthesia without epidural assistance; lithotomy positioning of the patient; four trocar placements in a horizontal line around the umbilicus (assistant port at the left end); 27° head-down positioning after trocar insertion; and robotic system roll-in and docking followed by console/cockpit operation.

**Figure 1:**
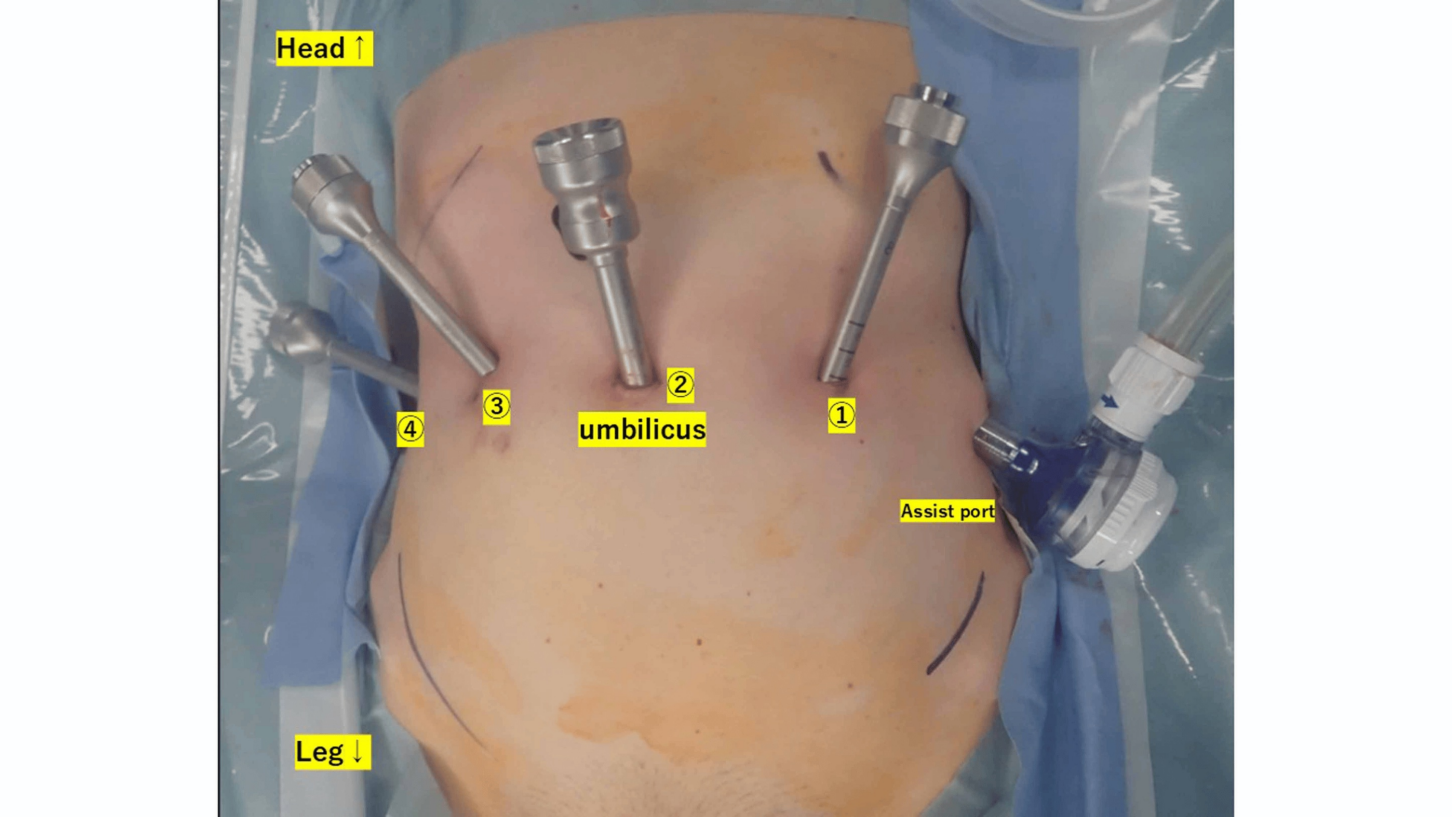
Port layout of the hinotori™ surgical robot system This image is an original from our study.

Figure 1 illustrates the port configurations.

Outcomes (O): Evaluation of Surgical Factors

Data collection and parameters assessed: Patient demographics, surgical details, and postoperative outcomes were collected from electronic medical records.

The preoperative evaluated variables included: age and body mass index (BMI)

The intraoperative evaluated variables included: total operative time; robotic surgery time; time from start of surgery to roll-in; time from roll-in to start of console/cockpit surgery; estimated blood loss; conversion to laparotomy; and intraoperative complications (bowel, bladder, ureteral, vascular injury).

The postoperative evaluated variables included: length of hospital stay (under Japan’s Diagnosis Procedure Combination (DPC) system) and rehospitalization rates within 30 days.

Postoperative complications were classified using the Clavien-Dindo system [6] as: Grade I: No intervention required; Grade II: Pharmacological treatment required; Grades III-IV: Surgical or intensive care required; Grade V: Death.

Statistical analysis

Categorical variables were analyzed using the chi-square test. Continuous variables were compared using the Wilcoxon rank sum test. A p-value < 0.05 was considered statistically significant. All analyses were conducted using JMP statistical software (version 14, SAS Institute Inc., Cary, North Carolina, US).

## Results

Table 2 compares clinical characteristics between the hinotori and the dVSS. A total of 17 patients underwent SH using the hinotori while 23 underwent the procedure with the dVSS. The median ages for the hinotori and dVSS groups were 48 and 47 years, respectively, and a significant variation in BMI was observed among the groups. No significant differences were observed between the two groups in terms of history of abdominal surgery, parity, or preoperative diagnosis. 

**Table 2:** Comparison of clinical characteristics between the hinotori™ SRS and da Vinci®

Characteristics	hinotori™ SRS (n = 17)	da Vinci® Xi (n = 23)	P-value
Median age (years)	48 (37–72)	47 (34–85)	0.90
Median BMI (kg/m^2^)	25.6 (17.3–33)	34 (21.8–45)	0.0004
History of abdominal surgery			0.63
No	13 (76%)	16 (70%)	-
Yes	4 (24%)	7 (30%)	
Parity			0.92
0	7 (41%)	8 (35%)	-
1	2 (12%)	2 (9%)	
2	3 (18%)	6 (26%)	
3	5 (29%)	7 (30%)	
Preoperative diagnosis			0.82
Uterine myoma	5 (29%)	7 (31%)	-
Uterine adenomyosis	3 (18%)	4 (17%)	
Endometrial hyperplasia	4 (24%)	7 (31%)	
Endometrial polyp	0 (0%)	1 (4%)	
Cervical intraepithelial carcinoma/dysplasia	5 (29%)	4 (17%)	

Table 3 shows a detailed comparison of surgical metrics between the hinotori and the dVSS. No statistically significant difference was found in operative time, cockpit or console time, or the duration from the start of surgery to roll-in for either system. However, the hinotori demonstrated a longer interval from roll-in to the beginning of console use (P = 0.024). Despite this, the hospital stay duration was notably shorter for patients in the hinotori™ group (P = 0.0015). Blood loss and complication rates were comparable, with the exception of one case of intraoperative bladder injury in the dVSS cohort, which was successfully addressed during surgery. Postoperative complications were observed in two cases, both of which involved postoperative pelvic infections. These cases were classified as Grade II complications, as they were successfully managed with intravenous antibiotic therapy without the need for surgical intervention. However, one of these cases experienced a delayed recovery from a pelvic infection, resulting in a prolonged hospital stay of 21 days.

**Table 3:** Comparison of surgical characteristics between the hinotori™ SRS and da Vinci®

Characteristics	hinotori™ SRS (n = 17)	da Vinci® Xi (n = 23)	P-value
Median operation time (min)	209 (range, 92−313)	236 (range, 97–318)	0.25
Median cockpit/console time (min)	153 (range, 60−225)	177 (range, 58–259)	0.22
Median time from operation start to roll-in (min)	16 (range, 8−29)	18 (range, 10–106)	0.53
Median time from roll-in to cockpit/console start (min)	15 (range, 9−27)	11 (range, 4–19)	0.024
Median blood loss (mL)	25 (range, 5−104)	20 (range, 5−390)	0.73
Median length of hospital stay (days)	4 (range, 4−9)	6 (range, 4−21)	0.0015
Conversion to open surgery			-
No	17 (100%)	23 (100%)	
Yes	0 (0%)	0 (0%)	
Intraoperative complications			0.38
No	17 (100%)	22 (96%)	-
Yes	0 (0%)	1 (4%)	
Postoperative complications			0.83
No	16 (94%)	22 (96%)	-
Yes	1 (6%)	1 (4%)	

## Discussion

This study assessed the surgical results of SH for benign uterine conditions performed by the hinotori as compared to the dVSS. Findings indicate that both systems provide comparable results in terms of median operation and cockpit or console times. However, the hinotori demonstrated a significantly longer setup duration from roll-in to the start of cockpit or console operations. Despite this, patients treated with the hinotori experienced significantly shorter hospital stays, suggesting potential advantages in postoperative recovery. Rates of conversion to laparotomy and incidences of intraoperative and postoperative complications were equivalent between the two systems.

A meta-analysis comparing robotic versus laparoscopic surgery for hysterectomy of benign uterine lesions reported no significant differences in the amount of blood loss, operation time, or conversion rate to open surgery [5]. Moreover, robotic surgery was shown to reduce hospitalization, the amount of blood loss, and postoperative complications compared to open surgery [5]. Similarly, Ghomi et al. highlighted that robotic-assisted hysterectomy not only achieves shorter operative durations compared to laparoscopic techniques but also demonstrates higher cost efficiency for surgeons performing over 45 cases annually [7].

Both the dVSS and the hinotori are integrated platforms with multiple robotic arms, featuring 3D high-resolution visualization and finger-clutch mechanisms for operation. However, a key distinction between the two systems lies in their docking mechanisms. While the dVSS requires trocars to be docked to the robotic arms, the hinotori™ system employs a free-docking mechanism, utilizing a pivot point as its fulcrum rather than docking the robotic arms directly to the trocars. Another notable difference is in the available instrumentation. Unlike the dVSS, the hinotori™ system currently lacks a vessel-sealing device similar to the vessel sealer, which may impact surgical efficiency and hemostasis. These technical differences could potentially lead to variations in surgical outcomes, warranting a comparative investigation of these two systems. In the present study, we systematically evaluated these differences, and our findings suggest that certain intraoperative factors may indeed be influenced by the robotic system employed. A couple of studies have reported comparisons of surgical outcomes between the hinotori and da Vinci robotic platforms in urological procedures [8,9]. In robot-assisted radical prostatectomy, it has been reported that operative time and console time were significantly longer in the hinotori group compared to the da Vinci group while no significant differences were observed in estimated blood loss, intraoperative complications, major postoperative complications, or length of hospital stay between the two groups [8]. Similarly, in robot-assisted partial nephrectomy, no significant differences were noted in operative time or console time between the two groups, and no perioperative complications were reported [9]. In the current study, median operative times for the hinotori and dVSS systems were 209 minutes and 236 minutes, respectively, with cockpit or console times of 153 minutes and 177 minutes. These values align with existing literature, including findings by Swenson et al. [10], which reported average operative durations of 138 ± 60 minutes for robotic procedures, and another report stated it was 142.96 ± 82.657 min [11], which is comparable to our study results. The roll-in to console initiation times for the hinotori and dVSS were 15 minutes and 11 minutes, respectively, with the hinotori showing a significantly longer duration (P = 0.024). This discrepancy is likely attributable to the system’s recent introduction and limited user experience, leading to inefficiencies during setup. Furthermore, it is necessary to consider the possibility that the higher BMI in the dVSS group may have influenced the results of this study. Matsuura et al. similarly observed variations in docking and console times across different robotic platforms, with the hinotori requiring longer durations compared to other models [12].

This study identified one case of intraoperative bladder injury in the dVSS group, which was promptly repaired. This rate aligns with findings by Petersen et al. [13], who reported a 0.92% incidence of urological injuries during robotic hysterectomy unaffected by patient BMI.

As of early 2024, the hinotori has been deployed in 45 units across two countries. Based on prior experience with 12 cases, the system has proven effective for minimally invasive gynecological surgeries [4]. Results from this study further confirm that the hinotori achieves outcomes comparable to the dVSS in SH for benign uterine diseases.

The limitations of this study include the small number of cases in both groups and the fact that it is a retrospective study. In addition, factors such as uterus size and surgeon experience were not evaluated, and the higher BMI in the dVSS group may have influenced the difference in hospitalization duration between the two groups.

## Conclusions

In conclusion, the hinotori offers an effective and safe alternative for robotic-assisted SH in benign uterine conditions. While setup times were longer for the hinotori, its shorter hospital stays highlight potential benefits. The hinotori is expected to gain increasing adoption both domestically and internationally in the coming years. However, aspects such as cost-effectiveness, which were not analyzed in the present study, remain important topics for future investigation.
